# Commentary: Health-Associated Niche Inhabitants as Oral Probiotics: The Case of *Streptococcus dentisani*

**DOI:** 10.3389/fmicb.2018.00340

**Published:** 2018-02-27

**Authors:** Georg Conrads, Julia A. Bockwoldt, Caroline Kniebs, Mohamed M. H. Abdelbary

**Affiliations:** Division of Oral Microbiology and Immunology, Department of Operative and Preventive Dentistry and Periodontology, RWTH Aachen University Hospital, Aachen, Germany

**Keywords:** *Streptococcus oralis* subspecies *dentisani*, probiotics, dental caries, pH buffering, arginolytic pathway, PCR detection

We comment on López-López et al. and their contributions on *Streptococcus dentisani*, especially the species-specific primers, buffering capacity, and prevalence in the oral cavity of healthy individuals (Camelo-Castillo et al., 2014; López-López et al., 2017), and its infective potential. *S. dentisani* was effectively reclassified as *Streptococcus oralis* subspecies *dentisani* by Jensen et al. (2016) and there exist two other closely related subspecies, *S. oralis* ssp. *oralis* and *S. oralis* ssp. *tigurinus* (Zbinden et al., 2012; Jensen et al., 2016; Conrads et al., 2017). López-López et al. studied the capacity of strains 7746 and 7747^T^ for buffering low pH and thus preventing overgrowth of acidogenic and aciduric cariogenic species such as *Streptococcus mutans*. In addition, they developed two PCRs to quantify *S. oralis* ssp. *dentisani* in clinical specimens, one directed against the carbamate kinase gene *arcC* and one against ORF540, the latter coding for a bacteriocin-related protein. Primer specificity was evaluated with DNA from *S. mutans, S. sobrinus, S. sanguinis, S. salivarius, S. mitis, S. pneumoniae, S. infantis*, and *S. oralis* ATCC 35037^T^. They conclude that *arcC*-primers did not amplify any of the related streptococcal species and ORF540-primers cross-reacted with *S. pneumoniae* only.

With the intention of screening our own strain collection for the presence of *S. oralis* ssp. *dentisani*, we tested these PCR primers with the type strains of *S. oralis* ssp. *dentisani* 7747^T^, ssp. *oralis* ATCC 35037^T^, ssp. *tigurinus* Az3a^T^, six *S. mitis* strains (OMI 181-184, 187-188) and four *S. pneumoniae* strains (OMI 157-159, 186). The *arcC*-directed PCR (Figure 1A, CkSd-F/R) was indeed very specific but the product was very short (76 bp) and we designed the alternative CkSdAlt-F/R primer pair amplifying a longer fragment of 175 bp (Figure 1A). The ORF540-directed PCR was found to be more bacteriocin-type than species-directed. Furthermore, we designed a 16S rRNA-gene-directed PCR expanding the options for application (Figure 1A, SDent-16S-F/R). Applying the two Ck- and the 16S-directed PCR, we screened our strain collection of clade *S. oralis* strains. We found four *S. oralis* ssp. *dentisani* strains (SN39325, SN54787, SN54788, SN58364) among isolates of proven and epidemiologically unrelated cases of infective endocarditis (National Reference Center for Streptococci, RWTH Aachen University Hospital, Germany; described in Conrads et al., 2017), and two strains (OMI 214 and OMI 215) isolated from the tooth surface of healthy caries-free probands. Next, we characterized these strains genetically by sequencing the 16S-rRNA gene (Figure 1B) and phenotypically *in-vitro* for their buffering capacity (Figure 1C). The buffering phenotype is dependent on the arginine deiminase system, which is determined by *arcA* (arginine deiminase), *arcB* (ornithine carbamoyltransferase), and *arcC* (carbamate kinase). The system is activated by a pH drop and leads to ammonia formation and neutralization of the environment and is—besides bacteriocin production—the reason for the probiotic and anti-cariogenic nature. López-López et al. tested the buffer capacity of strain 7746 only under aerobic conditions (A. Mira, personal communication). However, the atmospheric conditions in the oral cavity and especially on tooth surfaces are rarely truly aerobic, but instead something between micro-aerobic, CO_2_-rich, and anaerobic. Thus, we reproduced the growth-experiments in the same arginine-enriched BHI-medium and applying the same protocol but this time under aerobic, CO_2_-rich (7%), and anaerobic conditions. Together with our six *S. oralis* ssp. *dentisani* strains, we used *S. oralis* ssp. *dentisani* (7746, 7747^T^) and *S. gordonii* strains (OMI 231, GH 355) as positive controls of a functional arginine deiminase system. *S. oralis* ssp. *oralis* and ssp. *tigurinus*, as well as un-inoculated BHI (plus/minus arginine), served as negative controls. Clearly, all our six isolates demonstrated buffering activity, even more strongly pronounced than with the strains 7746 and 7747^T^ (Figure 1C). An increase of buffering activity was detected for *S. oralis* ssp. *dentisani* strains from CO_2_ (Δ pH 0.59–1.65, mean 1.36, median 1.43) to aerobic (Δ pH 0.56–2.22, mean 1.64, median 1.73) to anaerobic (Δ pH 1.41–1.85, mean 1.74, median 1.79) conditions. This increased activity under anaerobic conditions was significant for 5 of the 8 *S. oralis* ssp. *dentisani* strains tested. In time-curve experiments we confirmed that the alkalization process in arginine-supplemented BHI medium started between 6 and 8 h after inoculation but in the atmosphere of 7% CO_2_ it needed a pH as low as ≤ 5.5 to be induced. This finding has clinical impact since under microaerophilic, CO_2_-rich conditions, re-alkalization might start later than de-mineralization.

**Figure 1:**
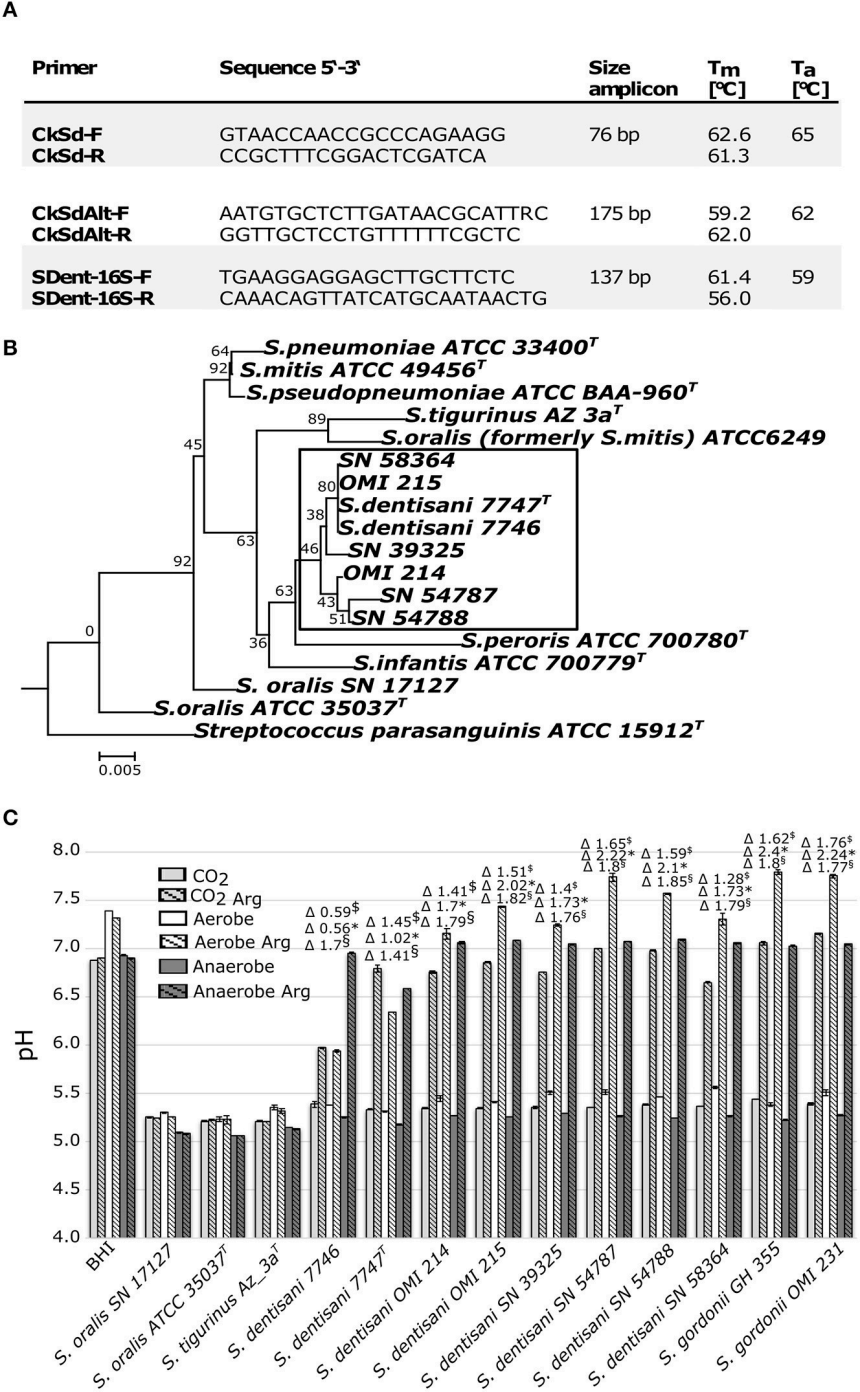
**(A)** Primer and conditions to specifically amplify *S. oralis* subspecies *dentisani*. **(B)** Neighbor-Joining phylogenetic tree based on the 16S rRNA-gene of 17 Mitis group streptococci including strains subjected in this study using MEGA6 with default settings and 200 bootstraps. SN-strains are endocarditis isolates of our collection. *S. parasanguinis* ATCC 15912^T^ was used as outgroup. **(C)** Endpoint pH values and Δ pH values of *S. oralis* subspecies *dentisani* and related (sub-)species after growth over 16 h in BHI with and without the addition of arginine monohydrate (Arg, 5 gl^−1^) and under 7% CO_2_, aerobic and anaerobic conditions. ^$^Δ pH 7% CO_2_, ^*^Δ pH aerobic, ^§^Δ pH anaerobic, BHI, Brain Heart Infusion medium control. The three *S. oralis* subspecies are abbreviated as *S. dentisani, S. oralis*, and *S. tigurinus*.

Finally, applying the *arcC*- and 16S-subspecies specific primers for real-time PCR-quantification, we subjected 10 saliva samples (1.5 ml freshly stimulated saliva processed to 100 μl DNA extract) of healthy, caries-free probands (aged 23–32, randomly selected from an ethically approved past study (Conrads et al., 2017). In principal, the *arcC*- and 16S-directed primers led to the same cell numbers which were between 1.2 × 10^1^ and 1.2 × 10^5^ cells per μl DNA extract or 8.0 × 10^2^ and 8.1 × 10^6^ per ml saliva. López-López et al. described much higher numbers (1.04 × 10^7^ and 6.94 × 10^7^) but they were derived from the pooled dental plaque of two individuals. Combined with the results of a universal bacterial 16S-directed PCR (modified from Nadkarni et al., 2002), we calculated the relative numbers of *S. oralis* ssp. *dentisani* within our saliva samples as between 0.01 and 10.46% (mean 1.73%, median 0.13%).

In conclusion, we support the findings of López-López et al. but with the following limitations: (i) the ORF450-directed primer showed low accuracy. We have designed alternative primers amplifying a longer *arcC*-fragment and a V1-V2-fragment of the 16S, expanding the applications; (ii) the induction and potential of alkalization of *S. oralis* ssp. *dentisani* is dependent on the atmosphere, with best results obtained under anaerobic conditions; (iii) as we found four endocarditis-associated strains in our collection and a Danish group recently even found six (Rasmussen et al., 2017), it must be indicated that some strains of *S. oralis* ssp. *dentisani* might bear the potential for causing infective endocarditis in immunocompromised patients. The challenge thus is to find—or produce—a strain with the most probiotic and the least infective potential.

## Author contributions

GC supervised the study, wrote the commentary, and is the corresponding author. JB performed experiments on the phenotypic characterization of *S. dentisani* isolates. CK performed experiments on the genotypic characterization of *S. dentisani* isolates and designed the PCR primers. MA checked genomic data and all results for plausibility and commented on and corrected the manuscript.

### Conflict of interest statement

The authors declare that the research was conducted in the absence of any commercial or financial relationships that could be construed as a potential conflict of interest.

## References

[B1] Camelo-Castillo A. Benitez-Paez A. Belda-Ferre P. Cabrera-Rubio R. Mira A. (2014). *Streptococcus dentisani* sp. nov., a novel member of the mitis group. Int. J. Syst. Evol. Microbiol. 64, 60–65. 10.1099/ijs.0.054098-0 24006481

[B2] Conrads G. Barth S. Möckel M. Lenz L. van der Linden M. Henne K. (2017). *Streptococcus tigurinus* is frequent among *gtfR*-negative *Streptococcus oralis* isolates and in the human oral cavity, but highly virulent strains are uncommon. J. Oral Microbiol. 9:1307079. 10.1080/20002297.2017.1307079 28473881 PMC5405715

[B3] Jensen A. Scholz C. F. Kilian M. (2016). Re-evaluation of the taxonomy of the Mitis group of the genus *Streptococcus* based on whole genome phylogenetic analyses, and proposed reclassification of *Streptococcus dentisani* as *Streptococcus oralis* subsp. *dentisani* comb. nov., *Streptococcus tigurinus* as *Streptococcus oralis* subsp. *tigurinus* comb. nov., and *Streptococcus oligofermentans* as a later synonym of *Streptococcus cristatus*. Int. J. Syst. Evol. Microbiol. 66, 4803–4820. 10.1099/ijsem.0.001433 27534397

[B4] López-López A. Camelo-Castillo A. Ferrer M. D. Simon-Soro Á. Mira A. (2017). Health-associated niche inhabitants as oral probiotics: the case of *Streptococcus dentisani*. Front. Microbiol. 8:379. 10.3389/fmicb.2017.00379 28344574 PMC5344910

[B5] Nadkarni M. A. Martin F. E. Jacques N. A. Hunter N. (2002). Determination of bacterial load by real-time PCR using a broad-range (universal) probe and primers set. Microbiology 148, 257–266. 10.1099/00221287-148-1-257 11782518

[B6] Rasmussen L. H. Højholt K. Dargis R. Christensen J. J. Skovgaard O. Justesen U. S. (2017). *In silico* assessment of virulence factors in strains of *Streptococcus oralis* and *Streptococcus mitis* isolated from patients with infective endocarditis. J. Med. Microbiol. 66, 1316–1323. 10.1099/jmm.0.000573 28874232

[B7] Zbinden A. Mueller N. J. Tarr P. E. Spröer C. Keller P. M. Bloemberg G. V. (2012). *Streptococcus tigurinus* sp. nov., isolated from blood of patients with endocarditis, meningitis and spondylodiscitis. Int. J. Syst. Evol. Microbiol. 62, 2941–2945. 10.1099/ijs.0.038299-0 22357776

